# Functions and Regulatory Mechanisms of lncRNAs in Skeletal Myogenesis, Muscle Disease and Meat Production

**DOI:** 10.3390/cells8091107

**Published:** 2019-09-19

**Authors:** Shanshan Wang, Jianjun Jin, Zaiyan Xu, Bo Zuo

**Affiliations:** 1 Key Laboratory of Swine Genetics and Breeding of the Ministry of Agriculture and Rural Affairs, Huazhong Agricultural University, Wuhan 430070, China; 2 Key Laboratory of Agriculture Animal Genetics, Breeding and Reproduction of the Ministry of Education, Huazhong Agricultural University, Wuhan 430070, China; 3 Department of Basic Veterinary Medicine, College of Veterinary Medicine, Huazhong Agricultural University, Wuhan 430070, China; 4 The Cooperative Innovation Center for Sustainable Pig Production, Wuhan 430070, China

**Keywords:** lncRNA, myogenesis, muscle disease, meat production

## Abstract

Myogenesis is a complex biological process, and understanding the regulatory network of skeletal myogenesis will contribute to the treatment of human muscle related diseases and improvement of agricultural animal meat production. Long noncoding RNAs (lncRNAs) serve as regulators in gene expression networks, and participate in various biological processes. Recent studies have identified functional lncRNAs involved in skeletal muscle development and disease. These lncRNAs regulate the proliferation, differentiation, and fusion of myoblasts through multiple mechanisms, such as chromatin modification, transcription regulation, and microRNA sponge activity. In this review, we presented the latest advances regarding the functions and regulatory activities of lncRNAs involved in muscle development, muscle disease, and meat production. Moreover, challenges and future perspectives related to the identification of functional lncRNAs were also discussed.

## 1. Introduction

Skeletal muscle is a heterogeneous organ composed of muscle fibers, the basement membrane, muscle satellite cells, and nerves [1]. In animal production, skeletal muscles are the main resources of animal protein for human consumption, and the growth and development of skeletal muscle directly influence animal meat quantity and quality. In medicine, abnormal regulation of skeletal muscle leads to many types of muscle disease, such as muscular atrophy, muscular dystrophy, muscular hypertrophy, and myosarcoma. Therefore, understanding the regulatory network of myogenesis could contribute to improved agricultural animal meat production, and to treating muscle diseases. During embryonic development, skeletal muscle originates from the myotome, and further forms myogenic progenitor cells under the regulation of the Shh, Notch and Wnt signaling pathways [2]. Muscle progenitor cells express paired box 3 (*Pax3*) and paired box 7 (*Pax7*) genes, and migrate to the limbs and trunk. *Pax3* and *Pax7* are upstream regulators that can induce expression of the myogenic factor 5 (*Myf5*) and myogenic differentiation 1 (*MyoD*), thus promoting the differentiation of muscle progenitor cells to myoblasts [3,4,5]. Overexpression of *Pax3* and *Pax7* can lead to excessive proliferation of myoblasts [6]. Skeletal myogenesis is an orderly process regulated by a series of muscle-specific transcription factors, including MyoD, myogenin (MyoG), Myf5, myogenic regulatory factor 4 (MRF4), and myocyte enhancer factor 2 (MEF2) [7,8]. Myogenesis requires these transcription factors to be expressed at the right time and location [9,10]. For example, *MyoD* overexpression converts fibroblasts into myoblasts and leads to the subsequent fusion into myotubes [11,12]. *MyoG* knockdown reverses terminal muscle cell differentiation [13,14]. In addition, myogenesis is also regulated by epigenetic modification. For example, myogenesis is accompanied by dynamic changes in global chromosome modification, especially histone modification in myogenic genes [15,16,17,18,19]. During postnatal muscle development, muscle satellite cells are divided into two groups; one of which continues to proliferate and differentiate to form new muscle fibers. The other group is stored in the basement membrane as muscle stem cells, which are quiescent under normal conditions [20,21]. Once the muscle is injured or stimulated, resting muscle satellite cells are activated immediately to express the *Pax7* gene. These satellite cells then begin to proliferate, migrate, and differentiate, fusing to form new muscle fibers to supplement the injured site [22,23]. Moreover, muscle regeneration is regulated by myogenic regulatory factors, the immune system, epigenetic modification, and the satellite cell microenvironment [17,19,22,24,25].

Although 80% of the eukaryotic genome is transcribed, only 2% of transcripts are translated into proteins [26]. Noncoding transcripts account for the vast majority of eukaryotic transcripts. The noncoding RNAs mainly include microRNAs (miRNAs), Piwi-interacting RNAs (piRNAs), circular RNAs (circRNAs), and long noncoding RNAs (lncRNAs). In this article, we focus primarily on lncRNAs, which are more than 200 nucleotides in length and have no protein-coding capacity [27]. Most lncRNAs are transcribed by RNA polymerase II, 5′ end capped, 3′ end poly(A) tailed, and post-transcription spliced [28,29,30]. Although lncRNAs are less abundant, less evolutionarily conserved, and have fewer exons compared to mRNA, their expression patterns are more spatio-temporally specific [31,32,33]. In the last decade, an increasing number of studies have indicated that lncRNAs participate in diverse cell and tissue development processes, such as X chromosome inactivation, genomic imprinting, stem cell maintenance, embryonic development, myogenesis, immunity, and tumorigenesis [34,35,36,37,38,39]. lncRNAs exert their functions through diverse mechanisms, including by regulating chromosome structures, gene transcription, mRNA stability and translation, and post-translational modification [32]. Here, we review recent advances regarding the importance of lncRNAs in skeletal muscle development, regeneration, and disease.

## 2. Functions and Mechanisms of lncRNAs in Muscle Development and Regeneration

**Figure 1:**
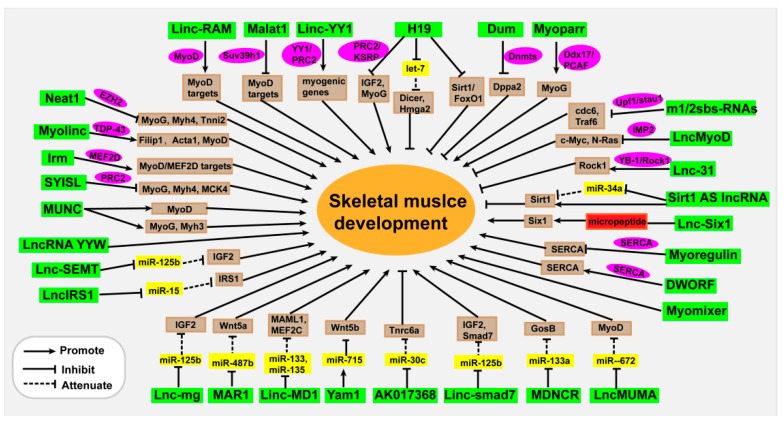
Functional lncRNAs in skeletal muscle development.

Thousands of lncRNAs have been detected in skeletal muscles. However, the function of most lncRNAs in muscle is still unclear, and only a small fraction of lncRNAs have been characterized. These lncRNAs exert functional roles through multiple mechanisms, including chromosome modification, transcription activation, molecular sponge activity, competitive binding, mRNA translation, and protein stability (Figure 1 and Table 1).

### 2.1. lncRNAs Regulate Chromosome Modification

lncRNA function is associated with their subcellular localization. Nuclear-retained lncRNAs play important roles in regulating gene transcription [33,87]. Nuclear lncRNAs can influence chromosome states by interacting with chromosome modification complexes, such as Polycomb Repressive Complex 2 (PRC2), and Switch/Sucrose nonfermentable (SWI/SNF) [88,89,90]. Some lncRNAs can regulate myogenesis by recruiting chromosome modification complexes to target gene promoters. Jin et al. (2018) identified and characterized a new lncRNA, *SYNPO2* intron sense-overlapping lncRNA (SYISL), involved in myoblast differentiation. Overexpression of SYISL significantly delays cell differentiation and promotes proliferation for C2C12 cells. Furthermore, knockout of SYISL significantly increases muscle mass and number of muscle fibers, and accelerates injury-induced muscle regeneration in vivo. Mechanistically, SYISL recruits PRC2 to the promoters of the target gene (e.g., *p21*, *MyoG*, or *myh4*), leading to H3K27me3 deposition [40]. Similarly, the *Neat1* lncRNA modulates myogenesis by recruiting PRC2 to epigenetic-silenced target genes [14]. lncRNA Malat1, which was discovered in cancer cells, can promote the proliferation of cancer cells and tumor progression [91,92]. Malat1 regulates myoblast differentiation and muscle regeneration by recruiting the Suv39h1 protein to the binding site of MyoD, resulting in trimethylation of lysine 9 of histone 3 (H3K9me3) at the binding site, inhibiting myogenic gene expression [41]. Moreover, lncRNAs also regulate myogenesis by detaching chromosome modification complexes from target gene promoters. Linc-YY1 is transcribed upstream of the *YY1* promoter and interacts with YY1. This interaction causes dissociation of the YY1/PRC2 complex from the promoters of the target gene, including *miR-29*, *miR-1*, *MyHC*, and *Troponin*, and reactivates their expression, promoting myoblast differentiation and regeneration [42].

### 2.2. lncRNAs Influence Transcription Activation

In addition to interacting with chromosome modification complexes, lncRNAs can also bind to transcription factors or RNA binding proteins to influence transcription activation. Linc-RAM, which is induced by MyoD, recruits MyoD to myogenic marker gene promoters to activate their transcription, thereby promoting muscle growth and regeneration [43]. Inhibition of Linc-RAM is essential for epidermal growth factor-related protein 2 (EGF2)-mediated suppression of myogenic differentiation [93]. An lncRNA called *Myolinc* is muscle-enriched and accelerates myogenesis by regulating its neighboring protein-coding gene, *Filip1 in cis,* and interacting with TAR DNA-binding protein 43 (TDP-43), a DNA/RNA-binding protein that regulates muscle-related gene expression (e.g., *Acta1* and *MyoD*) [45]. A lncRNA called *Myoparr*, which is expressed from the MyoG gene promoter region, can promote myoblast differentiation and inhibit myoblast proliferation. Myoparr is essential for increasing the interaction between Ddx17 and PCAF, and promotes binding of the Ddx17/PCAF complex to the *MyoG* promoter; then, it recruits Pol II to the *MyoG* promoter to further promote *MyoG* transcription and myoblast differentiation [46]. lncRNA Irm is upregulated upon myoblast differentiation and promotes myogenic differentiation and regeneration by directly binding to MEF2D and promoting the assembly of MyoD/MEF2D on the regulatory elements of target genes [47].

Enhancer RNAs (eRNAs) are a large class of lncRNAs that are transcribed from known DNA enhancer regions, and play important roles in transcriptional activation of neighboring genes by recruiting core transcription factors or accelerating the interaction between enhancers and promoters [94,95]. The myogenic eRNA *MyoD* upstream noncoding RNA (MUNC), also known as DRR^eRNA^, is reported to promote myoblast differentiation through at least two different mechanisms. First, MUNC acts as a typical eRNA to induce *MyoD* expression *in cis*. Second, MUNC can also act as an atypical eRNA to regulate *MyoG*, *Myh3*, and many other myogenic genes [48,49].

### 2.3. lncRNAs Serve as miRNA Molecular Sponges

The expression of lncRNA is highly associated with miRNA, suggesting that lncRNAs and miRNAs have co-regulatory functions in biological processes [96,97,98]. A lncRNA can act as a miRNA molecular sponge and weaken the inhibitory effects of miRNAs on target genes [99]. Many lncRNAs have been reported to regulate myogenesis by functioning as molecular sponges for miRNAs.

Linc-MD1 exhibits tissue-specific expression in skeletal muscle, and can promote the differentiation of skeletal muscle cells. During myogenic differentiation, linc-MD1 serves as a molecular sponge of miR-133 and miR-135 to attenuate the repression of their target genes, *MAML1* and *MEF2C*. This increases *MAML1* and *MEF2C* expression, thus promoting the differentiation of skeletal muscle [53]. LncRNA MAR1 is also highly expressed in muscle; overexpression of MAR1 can significantly promote myogenic differentiation and muscle growth. MAR1 can serve as a molecular sponge of miR-487b, weakening the effects of miR-487b upon its target gene, *Wnt5a*, and thereby promoting myogenic differentiation [54]. Lnc-mg is induced during myogenic differentiation and promotes myogenic differentiation and muscle regeneration by sponging miR-125b [56]. Lnc-mg can also regulate the expression of miR-351-5p, which can regulate myogenesis by targeting beta lactamase [57]. Linc-smad7 is a transcript of lncRNA-smad7 which has been reported to repress breast cancer cell apoptosis [100]. A transcriptome analysis of C2C12 cells demonstrated that Linc-smad7 is upregulated upon myoblast differentiation [58]. Overexpression of Linc-smad7 inhibits myoblast proliferation but promotes myoblast differentiation and regeneration. Linc-smad7 interacts with miR-125b and weakens the inhibitory effects of miR-125b upon its target genes, *IGF2* and *smad7* [59]. The lncRNA AK017368 promotes myoblast proliferation but inhibits myoblast differentiation by acting as a competing endogenous RNA of miR-30c [60,61].

### 2.4. lncRNAs Function at Post-Transcriptional Levels

Cytoplasm-located lncRNAs can regulate the expression of target genes at the post-transcriptional level by affecting the stability, splicing, and translation of mRNAs and the stability of proteins [101]. *m1/2sbs-RNA* is a type of lncRNA containing several SINE sequences that can complement genes containing the same SINE sequences, such as *Cdc6* and *Traf6*. It forms a STAU1-binding site (SBS), which leads to STAU1-mediated degradation of mRNA [63]. In addition to affecting the stability of mRNA, lncRNA can also regulate mRNA translation. LncMyoD, transcribed from the upstream region of the *MyoD* gene, can competitively bind the IMP2 protein. This reduces the binding ability of IMP2 for the target genes, *c-Myc* and *N-Ras*, inhibiting their translation and promoting cell differentiation [58]. Lnc-31 is a cytoplasmic long noncoding RNA that is downregulated during myoblast differentiation. Knockdown of lnc-31 expression inhibits myoblast proliferation but enhances myoblast differentiation [64]. Lnc-31 affects myogenesis by binding both Rock1 (a known myogenesis suppressor) mRNA and YB-1 (a translational regulator) protein, and promotes the positive effects of YB-1 on *Rock1* translation activation [65].

### 2.5. lncRNAs Encode Micropeptides

Although lncRNAs have little protein coding ability compared to mRNAs, some lncRNAs can give rise to functional micropeptides [102,103,104,105]. LncRNA-Six1 is located in the upstream of the protein-coding gene *Six1*, and has a role in promoting chicken skeletal muscle growth by regulating *Six1 in cis*. LncRNA-Six1 can produce a 7.26 kDa micropeptide, which plays an important role in lncRNA-Six1 cis-acting regulation of *Six1* [106]. Myoregulin (MLN) is a conserved micropeptide encoded by a skeletal muscle-specific putative lncRNA, and its expression is regulated by MyoD and MEF2. MLN binds directly to sarco-endoplasmic reticulum Ca^2+^ adenosine triphosphatase (SERCA), inhibiting SERCA activity and hindering the uptake of Ca^2+^ into the sarcoplasmic reticulum. Genetic deletion of MLN increases Ca^2+^ release in skeletal muscle, and improves muscle performance [66]. DWORF is a micropeptide of 34 amino acids encoded by a putative lncRNA that is specifically expressed in the heart and soleus. In mice, DWORF interacts with SERCA and increases SERCA activity, affecting muscle contraction. Knockout of DWORF in slow skeletal muscle leads to delayed Ca^+^ release and reduced SERCA activity [67]. Myomixer, also named Minion [70] and Myomerger [68], is an 84-amino acid muscle-specific micropeptide that interacts with Myomaker to promote cell fusion and skeletal muscle formation during embryogenesis [69]. A recent study demonstrated that Myomixer is also required for muscle regeneration [71]. The small regulatory polypeptide of amino acid response (SPAR) is encoded by the conserved lncRNA LINC00961. SPAR is located in the late endosome/lysosome and negatively regulates mTORC1 activation by binding to lysosomal v-ATPase in mammals. LINC00961 is highly expressed in skeletal muscle and is downregulated upon acute injury by CTX injection. Knocking out SPAR expression in mice while maintaining host lncRNA expression using CRISPR/Cas9 engineering significantly increases muscle regeneration after CTX injection by activating mTORC1, which has a positive effect on satellite cell proliferation, differentiation, and myofiber maturation [72,73].

Many lncRNAs have been shown to regulate myogenesis via multiple mechanisms. H19 can inhibit myoblast differentiation by recruiting PRC2 to the promoters of target genes [51,107,108], by serving as a molecular sponge of miRNA let-7 [74], or by recruiting KSRP protein to the 3′ end of MyoG mRNA to decrease the stability of MyoG mRNA [75]. Furthermore, H19 promotes muscle regeneration by producing two conserved miRNAs, miR-675-3p and miR-675-5p [76]. Another lncRNA, Sirt1 AS lncRNA, which is transcribed from the Sirt1 antisense strand, accelerates myoblast proliferation and represses myoblast differentiation by attenuating the inhibition of miR-34a to Sirt1 translation, and enhancing the stability of *Sirt1* mRNA [80].

## 3. lncRNAs in Skeletal Muscle Disease 

**Figure 2:**
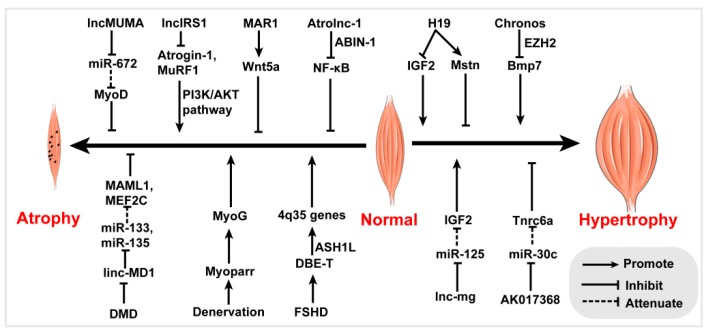
Functional lncRNAs involved in skeletal muscle disease.

Alterations in myogenesis and muscle regeneration may lead to numerous muscle diseases, such as sarcopenia, muscle hypertrophy, and muscular dystrophy. The abnormal expression of lncRNAs is associated with various muscle diseases, and rescue of their normal expression levels in skeletal muscle can alleviate the disease phenotype (Figure 2). Here, we have summarized the latest progress on lncRNAs in human muscle disease and animal muscular disease models.

### 3.1. LncRNAs in Human Skeletal Muscle Disease

Duchenne muscular dystrophy (DMD) is one of the most common and serious forms of muscular dystrophy, and is caused by losing functional dystrophin protein [109]. The DMD locus harbors multiple lncRNAs, and these lncRNAs repress the expression of the dystrophin mRNA isoforms through interacting with the *dystrophin* promoter [110]. Several lncRNAs exhibit expression changes in skeletal muscles in DMD patients relative to normal people. For example, lnc-31 is up-regulated in the skeletal muscles of DMD patients [64]. Linc-MD1 is down-regulated in myoblasts derived from muscles of DMD patients [53]. The myoblasts from DMD patients exhibit impaired cell differentiation, suggesting that the aberrant expression of lnc-31 and linc-MD1 is associated with DMD disease.

Facioscapulohumeral muscular dystrophy (FSHD) is the third most prevalent muscular dystrophy type, and results in progressive weakness and loss of skeletal muscles [111,112]. FSHD is linked to a reduction in the copy number of the 3.3 kb D4Z4 repeat mapping to 4q35, but is not associated with a classical mutation within a protein-coding gene. In FSHD patients, lncRNA DBE-T interacts with the Trithorax group protein Ash1L and recruits it to the FSHD gene locus, leading to H3K36me2 and de-repression of FSHD genes, and thus promoting FSHD pathogenesis [83].

The clinical symptoms of idiopathic inflammatory myopathies (IIM) include muscle weakness and inflammation (myositis). Next generation sequencing was employed to examine the transcriptome in muscle biopsies obtained from two histologically distinct patient populations: body myositis (IBM) patients and anti-Jo-1-associated myositis (Jo-1) patients. The results showed that 55 and 46 lncRNAs are differentially expressed in IBM and Jo-1 myositis patients compared to controls, respectively. Of these lncRNAs, 16 lncRNAs, including H19, lncMyoD and MALAT1, are differentially expressed in both IBM and Jo-1 myositis patients. These differentially expressed lncRNA may be involved in myositis [113].

### 3.2. LncRNAs in Skeletal Muscle Disease Models

#### 3.2.1. lncRNAs in Muscle Atrophy

Muscular dystrophy is the most common muscle disorder in humans, and is accompanied by muscle weakness and muscle wasting [114]. Several lncRNAs are differentially expressed between muscle dystrophy patients and normal individuals, including linc-MD1, lnc-31, Atrolnc-1, LncIRS1, and MAR1 [53,54,64,81,85]. Compared to normal cells, linc-MD1 expression levels are strongly reduced in Duchenne muscular dystrophy (DMD) myoblasts. Following overexpression of linc-MD1 in DMD myoblasts, the expression levels of *MyoG*, *MyHC, MEF2C* and *MAML1* return to normal [53].

Chronic kidney disease is commonly associated with cachexia, and causes skeletal muscle wasting. Sun et al. (2018) found that the expression of eight lncRNAs simultaneously increased in atrophying muscles in three mouse catabolic models, and nine lncRNAs were downregulated in atrophying muscles. One of the identified lncRNAs, Atrolnc-1, is abundantly expressed in skeletal muscle and its expression is markedly increased in atrophying muscles. Overexpression of Atrolnc-1 in muscle causes myofiber atrophy, while inhibition of Atrolnc-1 ameliorates muscle wasting in mice. Mechanistically, Atrolnc-1 strongly binds to ABIN-1, inhibiting NF-κB signaling and causing protein degradation in muscle cells [85].

LncIRS1 has also been identified as a regulator of muscle development, and can promote myogenic differentiation, muscle mass, and muscle cross-sectional area via sponging the miR-15 family to activate the IGF1-PI3K/AKT pathway. Importantly, in a dexamethasone-induced myotube atrophy model *in vitro*, lncIRS1 regulated the expression of muscle atrophy-related genes such as p-Foxo1, p-Foxo3, p-Foxo4, p-AKT, and Atrogin-1, and rescued dexamethasone-induced muscle atrophy in cultured myotubes [81].

The lncRNA MAR1 has been found to be downregulated in aged mice and mechanically unloaded mice. Enforced MAR1 expression attenuates muscle atrophy in mouse models of age-related muscle atrophy and mechanical unloading-induced muscle atrophy, suggesting that MAR1 could be a novel therapeutic target for treating muscle atrophy induced by aging or mechanical unloading [54]. MAR1 also affects myogenesis by enhancing Wnt5a function [54]. Wnt5a may contribute to age-related skeletal muscle atrophy in rats [55]. These studies suggest that MAR1 may be involved in the Wnt5a-regulated muscular atrophy pathway.

Another atrophy-related lncRNA, mechanical unloading-induced muscle atrophy-related lncRNA (lncMUMA), is the most downregulated lncRNA during muscle atrophy development in hindlimb suspension mice. LncMUMA promotes myogenesis by acting as an miR-762 molecular sponge to regulate *MyoD* expression. Therapeutically, the enforced expression of lncMUMA prevents muscle atrophy development and reverses established skeletal muscle atrophy following mechanical unloading [82].

The lncRNA Myoparr is transcribed from the upstream domain of the *MyoG* promoter, and promotes myogenic differentiation by regulating the association between Ddx17 and the histone acetyltransferase PCAF. Overexpression of Myoparr also promotes skeletal muscle atrophy caused by denervation, and knockdown of Myoparr rescues muscle wasting, suggesting that Myoparr may be a potential therapeutic target for neurogenic atrophy [46].

#### 3.2.2. lncRNAs in Muscle Hypertrophy

Muscle hypertrophy is associated with increased intracellular RNA and protein synthesis, and decreased protein degradation. The balance between protein synthesis and degradation is regulated by many pathways and regulators, such as the mTOR, IGF, and AMPK pathways, myostatin, and myogenic regulatory factors [115,116,117,118]. In addition, muscle hypertrophy requires activation of satellite cells [119,120]. Recent studies have indicated that several lncRNAs, such as H19, Chronos, lnc-mg and AK017368, are associated with muscle hypertrophy [56,60,77,79,87].

H19 is one of the earliest known examples of imprinted lncRNA, and plays a prominent role in regulating myogenic differentiation, which is fully repressed after birth except in skeletal muscle [77,121]. Deletion or mutation of H19 (H19^Δ3^) results in muscle hypertrophy and hyperplasia via reactivation of the imprinted gene network, particularly IGF2 upregulation following H19 deletion. Moreover, loss-of-function of H19 decreases myostatin (Mstn) expression [77,78,79]. These results suggest that H19 regulates muscle hypertrophy and hyperplasia mainly by influencing IGF2 and Mstn expression.

The Bmp7 signaling pathway positively regulates skeletal muscle hypertrophy through activation of Smad1/5 [122]. The muscle-enriched lncRNA Chronos is negatively regulated by Akt signaling and positively correlated with advancing age. Chronos epigenetically inhibits the expression of Bmp7 by recruiting EZH2. Knockdown of Chronos significantly increases the cross-sectional area of myofibers, and results in muscle hypertrophy in vivo [86].

## 4. Identification of lncRNAs in Agricultural Animal Meat Production

Muscle growth rate and muscle mass are two economically important traits in agricultural animal production. Compared with lncRNAs in model animals, the functions and mechanisms of lncRNAs affecting animal production are relatively unknown, although thousands of lncRNAs have been identified in livestock and poultry muscle (Table 2).

### 4.1. lncRNAs in Pig Skeletal Muscle Development

Tens of thousands of lncRNAs have been detected in the porcine genome through RNA sequencing (RNA-seq) and other technologies [133,134], most of which have been involved in pig skeletal muscle development [135,136,137,138,139,140]. Ren et al. (2009) isolated and identified the first pig lncRNA, trophoblast-derived noncoding RNA (*TncRNA*), which is differentially expressed in skeletal muscle in 90-day embryos of Tongcheng and Landrace pigs [139]. Zhao et al. (2015) identified more than 570 lncRNAs by systematically analyzing lncRNA expression in skeletal muscle at different times, and found an lncRNA, CUFF.8631, that is conserved among humans, mice, and pigs. This lncRNA contains four transcripts, and the transcripts CUFF.8631.1 and CUFF.8631.3 are differentially expressed during muscle development, suggesting that they may play a role in this process [140]. LncRNA MEG3 is differentially expressed in postnatal skeletal muscle development and conserved among humans, mice, and pigs. Four single nucleotide polymorphisms of MEG3 have been identified in Large White pigs and are associated with meat-producing traits [124].

### 4.2. lncRNAs in Bovine Skeletal Muscle Development

About 8000 lncRNAs expressed in bovine muscle have been identified and analyzed [141,142,143]. Several lncRNAs have been reported to play important roles in bovine myoblast proliferation and differentiation. For example, lncMD promotes bovine myoblast differentiation by acting as a molecular sponge of miR-125b [125]. Lnc133b regulates bovine skeletal muscle satellite cell proliferation and differentiation by sponging miR-133b [126]. LncRNA MDNCR promotes bovine myoblast differentiation but inhibits proliferation by acting as a molecular sponge of miR-133a, and thus weakens the inhibitory effects of miR-133a upon its target gene, *GosB* [127]. LncRNA H19 promotes bovine skeletal muscle satellite cell differentiation by repressing Sirt1/FoXO1 [128]. LncRNA MEG3 has a functional role in promoting bovine skeletal differentiation by sponging miR-135, attenuating the suppressive effects of miR-135 upon MEF2C [123]. LncRNA YYW is highly expressed in muscle, and promotes bovine myoblast proliferation and differentiation [129]. The lncRNA lncKBTBD10 is also induced during myogenic differentiation and plays a role in bovine skeletal muscle myogenesis [130].

### 4.3. lncRNAs in Sheep and Goat Skeletal Muscle Development

Zhan et al. (2016) identified 3981 lncRNAs in goat muscle tissues at different embryonic stages and three days after birth by RNA-seq, of which 577 lncRNAs were differentially expressed among the different stages of muscle development [144]. Ren et al. (2017) used Ribo-Zero RNA-seq technology to analyze the muscle lncRNA of Hu sheep at the fetal, lamb, and adult stages, and identified 6924 differentially expressed lncRNAs. GO analysis revealed that these differentially expressed lncRNAs are involved in muscle development and organ formation [145]. Li et al. (2019) identified 404 differentially expressed lncRNAs in sheep muscle from the prenatal to postnatal developmental stages using RNA-seq [146]. Lnc-SEMT, which is specifically expressed in muscle, can regulate IGF2 expression by sponging miR-125b, thus promoting muscle growth and development. Lnc-SEMT transgenic sheep exhibit significant muscle hypertrophy and weight gain [131].

### 4.4. lncRNAs in Chicken Skeletal Muscle Development

A total of 8072 chicken skeletal muscle-related lncRNAs have been detected by RNA-seq [147,148,149,150,151]. Among them, lncRNA-Six1 has a functional role in regulating chicken muscle development by sponging miRNA [132], and by encoding a micropeptide [106]. Another lncRNA, lncIRS1, is involved in regulating chicken muscle atrophy by acting as a molecular sponge for the miR-15 family to activate the IGF1-PI3K/AKT pathway [81].

## 5. Challenges and Future Perspectives

All of the above studies have shown that lncRNAs regulate multiple aspects of skeletal muscle development and disease by various regulatory mechanisms. Although the functions and mechanisms of some lncRNAs have been clearly studied, the research of lncRNAs in skeletal muscle is far from complete. Current studies regarding lncRNAs in muscles mainly focus on their roles in muscle atrophy and hypertrophy, muscle growth, and development after birth. Further attention should be paid to the regulation of muscle development during the embryonic stage, conversion of different types of muscle fibers, muscle aging, muscle metabolism, and muscle tumors. In addition, due to large number of lncRNA transcripts and low sequence conservation, the functions and mechanisms of lncRNAs are more complex than those of protein-coding genes. Therefore, there are still many unsolved problems and challenges ahead, including the following:

- As tens of thousands of lncRNAs have been identified in muscles, their functions should be further explored by high-throughput methods. Recently, the development of genome editing techniques such as CRISPR/Cas9 system has provided powerful tools to identify functional lncRNAs in vivo and in vitro [152,153,154]. Thus, construction of sgRNA library targeting lncRNAs and establishment of efficient screening systems for muscle cells will be beneficial to the screening of key functional lncRNAs in skeletal muscles.
- Continual innovation in data analysis tools has accelerated the investigation and identification of lncRNAs in myogenesis [155]. The development of computer models and algorithms provides an important basis for the functional prediction of lncRNAs [156,157,158,159]. Several databases, such as LncATLAS, starBase v2.0, CatRAPID, and RPISeq, have been established to predict the functions of lncRNAs, such as subcellular location, binding proteins, and miRNAs [160,161,162,163]. The computer aided functional characterizations of lncRNAs need to be further verified by experiments, as computer model-assisted predictions are mainly based on probability and statistics. Moreover, the annotation information in these databases is still incomplete, especially information relating to different transcripts. Non-poly (A) or other forms of lncRNAs, such as sno lncRNAs, are often ignored, as RNA-seq technology is mainly based on poly (A) sequencing techniques. Therefore, developing more advanced RNA-seq technologies and corresponding analysis tools will help us to recognize lncRNAs more comprehensively.
- lncRNAs can play regulatory roles by interacting with DNA, RNA and proteins, and systematic identification of molecules interacting with lncRNAs is essential to elucidating their molecular mechanisms of action. Thus, more efficient techniques such as ChIRP (Chromatin isolation by RNA purification) and dChIRP (domain-specific ChIRP) should be further developed to study lncRNA interactomes.
- lncRNAs can serve as biomarkers and therapeutic targets of several diseases, such as cancer, cardiopathy, neurologic diseases, and immunological diseases [164,165,166,167,168]. However, few lncRNAs have been identified and used as therapeutic targets for skeletal muscle diseases. Therefore, identifying more key lncRNAs related to skeletal muscle diseases will contribute to the treatment of skeletal muscle diseases in the future.

In this review, we presented the latest advances in the regulation network of lncRNAs in skeletal muscle development and muscle diseases, as well as the recent progress in agricultural animal meat production. Moreover, challenges and future perspectives were also discussed in the identification of novel muscle-related lncRNAs. Since the methods of studying lncRNAs have been reviewed in many studies, this review does not cover this aspect in much detail. In summary, lncRNAs play key roles in muscle development and regeneration, and in muscle diseases. The development of new tools and technologies will enable more functional lncRNAs to be identified in the future. Further studies will help to achieve an in-depth understanding of the functions and mechanisms of lncRNAs, and ultimately lead to the application of lncRNAs as therapeutic targets for muscle diseases or biomarkers for animal production.

## Figures and Tables

**Table 1:** Functional lncRNAs in skeletal muscle development and muscle disease.

LncRNAs	Location	Function	Mechanism	Muscle Disease	Ref.
SYISL	Nucleus/ Cytoplasm	Promotes proliferation, inhibits differentiation and muscle regeneration	Interacts with PRC2	Unknown	[40]
Neat1	Nucleus	Promotes proliferation and regeneration, inhibits differentiation	Interacts with EZH2	Unknown	[14]
Malat1	Nucleus	Inhibits differentiation and regeneration	Interacts with Suv39h1	Unknown	[41]
Linc-YY1	Nucleus	Promotes differentiation and regeneration	Interacts with YY1/PRC2	Unknown	[42]
Linc-RAM	Nucleus/ Cytoplasm	Promotes muscle growth and regeneration	Interacts with MyoD	Unknown	[43]
Dum	Nucleus/ Cytoplasm	Promotes differentiation and regeneration	Interacts with Dnmts	Unknown	[44]
Myolinc	Nucleus	Promotes differentiation and regeneration	Interacts with TDP-43	Unknown	[45]
Myoparr	Nucleus	Inhibits proliferation, promotes differentiation	Interacts with Ddx17/PCAF	Muscle atrophy	[46]
Irm	Nucleus	Promotes differentiation and regeneration	Interacts with MEF2D	Unknown	[47]
MUNC	Nucleus	Promotes differentiation	Induces MyoD, MyoG, Myh3 expression	Unknown	[48,49]
Meg3	Nucleus	Promotes skeletal development during embryogenesis	Interacts with PRC2	Unknown	[50,51]
SRA	Nucleus	Promotes differentiation	Assembly of p68/p72/MyoD coregulators	Unknown	[52]
Lnc-MD1	Cytoplasm	Promotes differentiation	MiR-133, miRNA-135 molecular sponge	Muscle atrophy	[53]
MAR1		Promotes differentiation and muscle growth	MiR-487b molecular sponge	Muscle atrophy	[54,55]
Lnc-mg	Nucleus/ Cytoplasm	Promotes differentiation and regeneration	MiR-125b molecular sponge	Muscle hypertrophy	[56,57]
Linc-smad7	Nucleus/ Cytoplasm	Inhibits proliferation, promotes differentiation and regeneration	MiR-125b molecular sponge	Unknown	[58,59]
AK017368	Nucleus/ Cytoplasm	Promotes proliferation, inhibits differentiation	MiR-30c molecular sponge	Muscle hypertrophy	[60,61]
Yam1	Nucleus/ Cytoplasm	Inhibits differentiation	Activates miR-715 expression	Unknown	[62]
m1/2sbs-RNAs	Cytoplasm	Regulates myogenesis	STAU1-mediated degradation of mRNA	Unknown	[63]
LncMyoD	Nucleus/ Cytoplasm	Promotes differentiation	Competitively binds to IMP2 protein	Unknown	[58]
Lnc-31	Nucleus/ Cytoplasm	Promotes proliferation, inhibits differentiation	Interacts with ROCK1/YB-1	Muscle atrophy	[64,65]
Myoregulin	SR/ER membrane	Reduces muscle performance	Binds to SERCA and inhibits its activity	Unknown	[66]
DWORF	SR membrane	Improves muscle contraction capacity	Binds to SERCA and increases its activity	Unknown	[67]
Myomixer	Membrane	Promotes fusion and regeneration and muscle formation during embryogenesis	Interacts with Myomaker	Unknown	[68,69,70,71]
LINC00961	Endosome/ Lysosome	Inhibits muscle regeneration	Interacts with the lysosomal v-ATPase	Unknown	[72,73]
H19	Nucleus/ Cytoplasm	Regulates differentiation and regeneration	Interacts with PRC2 or KSRP, miR-let7 molecular sponge, encodes miR-675	Muscle hypertrophy	[51,74,75,76,77,78,79]
Sirt1 AS lncRNA	Nucleus/ Cytoplasm	Promotes proliferation, inhibits differentiation	MiR-34a molecular sponge, stabilizes Sirt1 mRNA	Unknown	[80]
LncIRS1	Nucleus/ Cytoplasm	Promotes proliferation and differentiation	MiR-15 molecular sponge	Muscle atrophy	[81]
LncMUMA		Promotes differentiation	MiR-672 molecular sponge	Muscle atrophy	[82]
DBE-T	Nucleus	De-repressed muscle dystrophin mRNA isoforms	Interacts with ASH1L protein	Muscle atrophy	[83,84]
Atrolnc-1		Promotes muscle wasting	Interacts with ABIN-1	Muscle atrophy	[85]
Chornos		Inhibits muscle hypertrophy	Interacts with EZH2	Muscle hypertrophy	[86]

**Table 2:** LncRNAs involved in agriculture animal muscle development.

LncRNA	Location	Function	Mechanism	Ref.
MEG3	Mainly in cytoplasm	Promotes bovine myoblast differentiation; involved in pig meat production traits	MiR-135 molecular sponge	[123,124]
LncMD	Mainly in nucleus	Promotes bovine myoblast differentiation	MiR-125b molecular sponge	[125]
Lnc133b	Mainly in nucleus	Regulates bovine skeletal muscle satellite cell proliferation and differentiation	MiR-133b molecular sponge	[126]
MDNCR		Promotes bovine myoblast differentiation, inhibits cell proliferation	MiR-133a molecular sponge	[127]
H19	Nucleus/Cytoplasm	Promotes bovine skeletal muscle satellite cell differentiation	Represses Sirt1/FoxO1	[128]
YYW	Mainly in cucleus	Promotes bovine myoblast proliferation and differentiation		[129]
LncKBTBD10	Mainly in cucleus	Involved in bovine skeletal satellite cell proliferation and differentiation		[130]
Lnc-SEMT		Promotes sheep myoblast differentiation and muscle growth	MiR-125b molecular sponge	[131]
LncRNA-Six1	Nucleus/Cytoplasm	Promotes chicken myoblast proliferation and differentiation, and involved in skeletal muscle fiber types transformation	MiR-1611 molecular sponge	[132]
LncIRS1	Nucleus/Cytoplasm	Promotes the proliferation and differentiation of chicken myoblast	MiR-15 molecular sponge	[81]
